# Complete mitochondrial genome of *Rhizosolenia setigera* (Coscinodiscophyceae, Bacillariophyta)

**DOI:** 10.1080/23802359.2021.1950059

**Published:** 2021-07-12

**Authors:** Yanxin Yao, Feng Liu, Nansheng Chen

**Affiliations:** a CAS Key Laboratory of Marine Ecology and Environmental Sciences, Institute of Oceanology, Chinese Academy of Sciences, Qingdao, China; b Laboratory of Marine Ecology and Environmental Science, Qingdao National Laboratory for Marine Science and Technology, Qingdao, China; c School of Earth and Planetary, University of Chinese Academy of Sciences, Beijing, China; d Center for Ocean Mega-Science, Chinese Academy of Sciences, Qingdao, China; e Department of Molecular Biology and Biochemistry, Simon Fraser University, Burnaby, Canada

**Keywords:** Diatoms, mitochondrial genome, *Rhizosolenia setigera*

## Abstract

*Rhizosolenia* is a species-rich genus with 144 described species, many of which are harmful algal species (HABs) with significant negative ecological impact. Despite their significance in primary production and their potential to induce HABs, genome data of these species remain extremely limited. In this study, the complete mitochondrial genome (mtDNA) of *Rhizosolenia setigera* Brightwell 1858 was determined for the first time, which also represented the first mtDNA of the order Rhizosoleniales. The circular mtDNA was 34,792 bp in length with GC content of 23.28%. It encoded 63 genes including 35 protein-coding genes (PCGs), 24 transfer RNA (tRNA) genes, 2 ribosomal RNA (rRNA) genes, and 2 conserved open reading frames (*orf*s). Phylogenetic analysis using concatenated PCGs revealed that *R. setigera* and *Melosira undulate*, which also belongs to the class Coscinodiscophyceae, clustered together as expected. However, comparison of these two mtDNAs revealed extensive genome rearrangement events, suggesting large evolutionary distance. The complete mtDNA of *R. setigera* will facilitate research on the phylogenetic relationship among *Rhizosolenia* species, which will in turn facilitate exploration of the evolutionary relationships in the class of Coscinodiscophyceae.

*Rhizosolenia* (Coscinodiscophyceae, Bacillariophyta) is an important group of marine phytoplankton, which includes 144 species described globally (Guiry and Guiry 2020) and 17 species described in China (Qian and Chai 1989). *Rhizosolenia* is mostly abundant in mat formation containing communities of multiple *Rhizosolenia* species (Singler and Villareal 2005). *Rhizosolenia* mats are vertical migrators to transport nutrients from the bottom to the top in the oligotrophic ocean (Singler and Villareal 2005). *Rhizosolenia* plays a significant role in carbon, silica and nitrogen cycles in the oligotrophic seas ( Shipe et al. 1999; Pilskaln et al. 2005). Most researches on *Rhizosolenia* species focused on morphological features (Sorhannus 1990; Yun and Lee 2015), while molecular research on *Rhizosolenia* species remain insufficient. Here, we constructed the complete mtDNA of the species *Rhizosolenia setigera* Brightwell 1858. The strain CNS00456 analyzed in this study was isolated in water samples collected during an expedition to the Changjiang Estuary (31°39.722′N, 122°99.744′E) in August 2020 on board the research vehicle ‘Zheyuke 2’. Genomic DNA was extracted from the collection of marine algae *R. setigera* strain CNS00456. The DNA and specimen were deposited at KLMEES (Key Laboratory of Marine Ecology and Environmental Science) of IOCAS (Institute of Oceanology of the Chinese Academy of Sciences) (Nansheng Chen, chenn@qdio.ac.cn) under the voucher number CNS00456.

Illumina sequencing results of *R. setigera* genomic DNA were assembled into scaffolds using Spades v3.14.0 (Bankevich et al. 2012). Target scaffolds of mtDNA were selected from the assembly results using BLASTN v2.10.1, and *Toxarium undulatum* mtDNA as query. The annotation of PCGs was performed by using SmartBLAST (https://blast.ncbi.nlm.nih.gov/smartblast/) and BLASTP. Open reading frames (*orf*s) in the mtDNA were identified using Open Reading Frame Finder (ORF finder) (https://www.ncbi.nlm.nih.gov/orffinder). The locations of tRNA genes and rRNA genes were annotated using tRNAscan-SE 2.0 (Chan and Lowe 2019) with default setting and MFannot (https://megasun.bch.umontreal.ca/RNAweasel/).

The complete mtDNA of *R. setigera* (GenBank accession number: MW392567) was 34,792 bp in size, which was compact and smaller than most diatom mtDNAs. It had an overall GC content of 23.28%, which was substantially lower than that of the mtDNAs of *Skeletonema marinoi* (29.7%) (An et al. 2017) and *Pseudo-nitzschia multiseries* (31.04%) (Yuan et al. 2016), but it was slightly higher than that of *Melosira undulate* (21.6%) (Pogoda et al. 2019). It encoded 63 genes including 35 PCGs (*atp6*,*8*,*9*; *cob*, *cox1-3*; *nad1-7*,*4L*,*9*,*11*; *rpl2*,*5*,*6*,*14*,*16*; *rps2-4*,*7*,*8*,*10-14*,*19*; *tatA*,*C*), 24 transfer RNA (tRNA) genes, 2 ribosomal RNA (rRNA) genes, and 2 open reading frames (*orf*s). The start codons of all PCGs were ATG. No introns were identified in this mtDNA. The intergenic regions ranged from 0 bp to 426 bp in sizes, with an average size of 61 bp, which were similar to those of *Melosira undulate*, whose intergenic regions ranged from 2 bp to 530 bp in sizes, with an average size of 59 bp.

Phylogenetic analysis was implemented by utilizing 31 PCGs (*atp6*, *8*, *9*; *cob*; *cox1*, *2*, *3*; *nad1*-*7*, *4 L*, *9*, *11*; *rpl2*, *5*, *6*, *14*, *16*; *rps3*, *4*, *8*, *10*, *11*, *13*, *14*, *19*; and *tatC*) that were shared by *R. setigera* mtDNA and mtDNAs of other 36 diatom species (Figure 1). Maximum likelihood phylogenetic tree was constructed using IQtree v1.6.12 (Trifinopoulos et al. 2016) with 1000 bootstraps. mtDNA of one Parmales species *Triparma laevis* (AP014626) (Tajima et al. 2016) was used as out-group taxa. The phylogenetic tree showed that *R. setigera* mtDNA (MW392567) and *Melosira unduata* mtDNA (NC037728) clustered together within the class Coscinodiscophyceae. The length of *R. setigera* mtDNA was longer than that of *M*. *unduata* mtDNA, which is 32,777 bp (Pogoda et al. 2019). Compared with *M*. *unduata* mtDNA, *R. setigera* mtDNA contained an extra gene *rps2*. Colinearity analysis of the mtDNAs of *M*. *unduata* and *R. setigera* revealed extensive genome rearrangement events. For example, the relative position of *tatA* and *tatC* is different between these two species, consistent with their distant evolutionary relationship. *R. setigera* mtDNA represented the first mtDNA of the order Rhizosoleniales. More mtDNAs of this order are needed to study their phylogenetic relationships.

**Figure 1.**
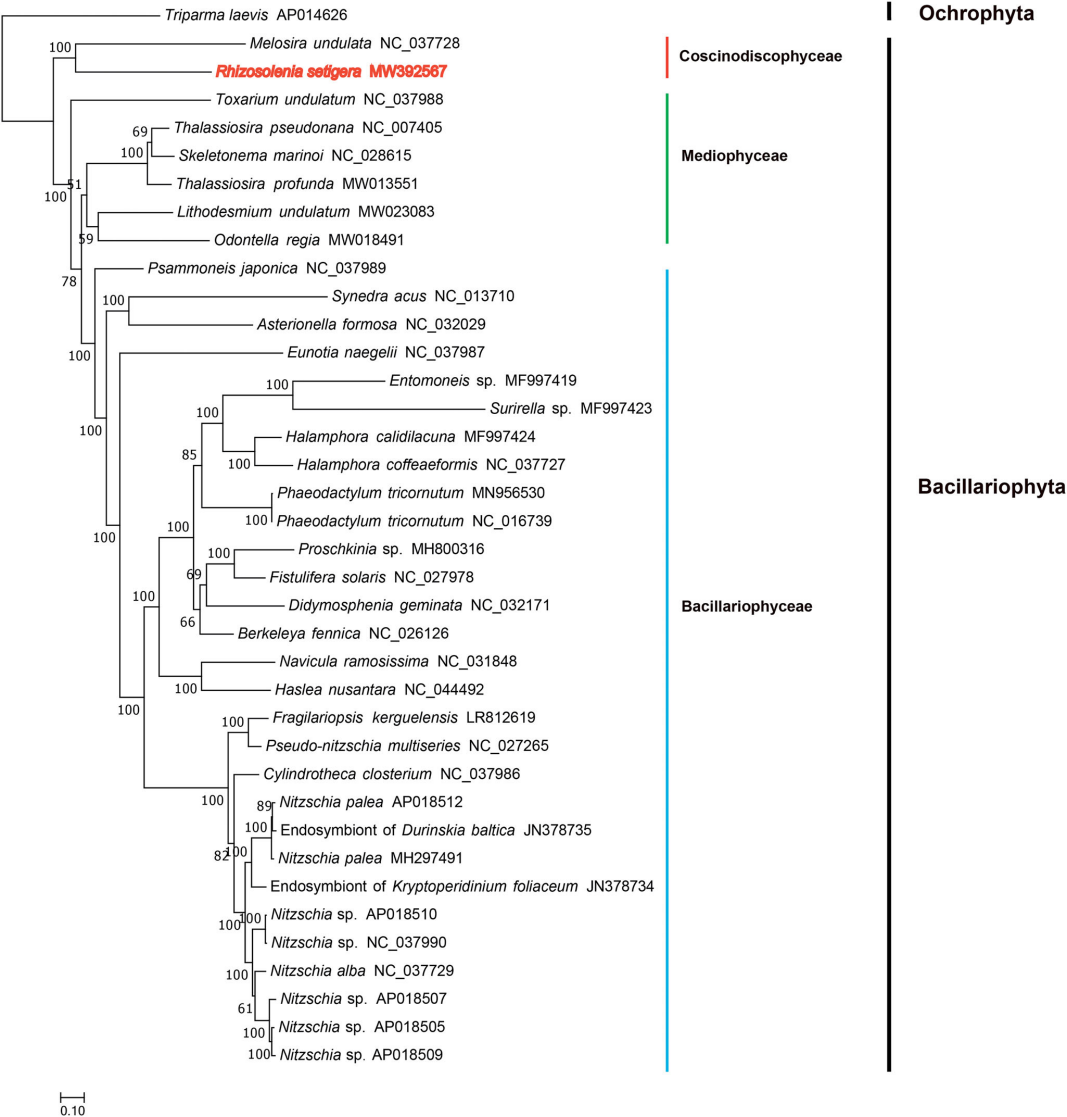
Maximum likelihood (ML) phylogenetic tree based on 31 concatenated PCGs from 36 publicly diatom mtDNAs, and mtDNA of one Parmales species *Triparma laevis* (AP014626) was used as out-group taxa. The numbers beside branch nodes were the percentage of bootstrap values.

## Data Availability

The genome sequence data that support the findings of this study are openly available in GenBank of NCBI at https://www.ncbi.nlm.nih.gov/under the accession number MW392567. The associated BioProject and Bio-Sample numbers are PRJNA686853 and SAMN17126607, respectively.
